# Atypical presentation of Epstein–Barr virus infectious mononucleosis with bilateral dacryoadenitis in an elderly patient: a case report

**DOI:** 10.1186/s13256-026-06165-x

**Published:** 2026-05-26

**Authors:** Nour Mahfoud, Mhammad Ali, Mohammad Abboud

**Affiliations:** 1 Faculty of Medicine, Al Andalus University for Medical Sciences, Tartus, Syria; 2 Faculty of Dentistry, Al Andalus University for Medical Sciences, Tartus, Syria; 3 Faculty of Medicine, Damascus University, Damascus, Syria

**Keywords:** Epstein–Barr virus, Infectious mononucleosis, Dacryoadenitis, Case report

## Abstract

**Background:**

Epstein–Barr virus (EBV) infection is a highly prevalent condition and the primary cause of infectious mononucleosis (IM), typically affecting adolescents and young adults. Although IM is generally self-limited, EBV may involve multiple organ systems, presenting with atypical or localized manifestations. Ocular involvement is uncommon and includes conditions, such as conjunctivitis, uveitis, and, more rarely, dacryoadenitis. Acute dacryoadenitis remains an infrequent manifestation of EBV infection and is most commonly reported in younger patients; its occurrence in elderly patients is particularly unusual.

**Case presentation:**

A 71-year-old woman presented with a 4-week history of bilateral upper eyelid edema following an episode of tonsillitis and cervical lymphadenopathy. Initial treatment with antibiotics and corticosteroids resulted in transient improvement, followed by relapse. Clinical examination revealed bilateral upper eyelid swelling with erythema, tearing, and S-shaped deformity, suggestive of lacrimal gland involvement. Laboratory investigations were unremarkable. As EBV infection was not included in the initial differential diagnosis, heterophile antibody testing was not obtained. Magnetic resonance imaging confirmed bilateral lacrimal gland enlargement without evidence of mass or infiltrative disease; whereas computed tomography imaging demonstrated unremarkable paranasal sinuses, with no significant abnormalities. Serological testing revealed positivity for Epstein–Barr virus viral capsid antigen IgM and IgG, consistent with recent infection. Given the transient response to short-term corticosteroids and select studies demonstrating the efficacy of prolonged therapy, oral prednisone was initiated at 1 mg/kg/day for the first week, followed by a three-week taper, resulting in rapid clinical improvement and complete resolution of symptoms. No recurrence was observed at 6-month follow-up.

**Conclusions:**

This case expands current knowledge of Epstein–Barr virus infectious mononucleosis by demonstrating an atypical presentation with bilateral dacryoadenitis in an elderly patient. The clinical course underscores the challenges in distinguishing viral from bacterial etiologies and highlights the importance of accurate diagnosis to guide management, as corticosteroid therapy may be beneficial in selected cases.

## Background

Epstein–Barr virus (EBV) is a highly prevalent herpesvirus and the primary cause of infectious mononucleosis (IM), particularly in adolescents and young adults. The disease typically results from delayed viral acquisition rather than early childhood infection. Consequently, it is predominantly observed in adolescents and young adults, and less commonly in preadolescents [1].

Symptomatic primary infection typically presents with fever, pharyngitis, cervical lymphadenopathy, marked fatigue, and atypical lymphocytosis [2]. Although most cases are self-limited, splenomegaly, hepatitis, and rare complications, including splenic rupture, may occur [3]. Almost any organ can be involved in EBV infection, and IM may begin with atypical or localized symptoms. Periorbital or palpebral edema is seen in up to one-third of IM patients and may reflect underlying lacrimal gland involvement. EBV IM can produce a wide range of ocular manifestations such as conjunctivitis, dacryocystitis, uveitis, and in rare cases, dacryoadenitis [4, 5].

Epstein–Barr virus genomes and antigens have been detected in the lacrimal glands of patients with Sjögren’s syndrome, suggesting a potential role in chronic lacrimal lymphoproliferation and gland destruction. This mechanism is distinct from the acute, self-limited dacryoadenitis observed during primary EBV infectious mononucleosis [6]. Dacryoadenitis is an uncommon but recognized manifestation of primary Epstein–Barr virus infection and infectious mononucleosis (IM). It typically presents with upper eyelid swelling and tenderness, along with imaging evidence of lacrimal gland enlargement, often in association with systemic IM features and EBV-positive serology [3]. This case highlights a rare presentation of Epstein–Barr virus infectious mononucleosis associated with dacryoadenitis in an elderly female patient, representing an exceptionally uncommon occurrence of primary EBV infection in this age group, and contributes to the limited literature on atypical EBV manifestations in this population.

## Case presentation

A 71-year-old woman was referred with a 4-week history of bilateral upper eyelid edema. She had no significant past medical history and reported no known allergies to foods or medications. The patient reported that the eyelid edema began following a prior episode of tonsillitis, which was accompanied by bilateral cervical lymph nodes enlargement. At that time, the condition was clinically diagnosed as lymphadenopathy-associated infection. She was treated with a 2-week course of oral antibiotics and corticosteroids, resulting in initial improvement, until relapse occurred without sustained recovery.

**Fig. 1:**
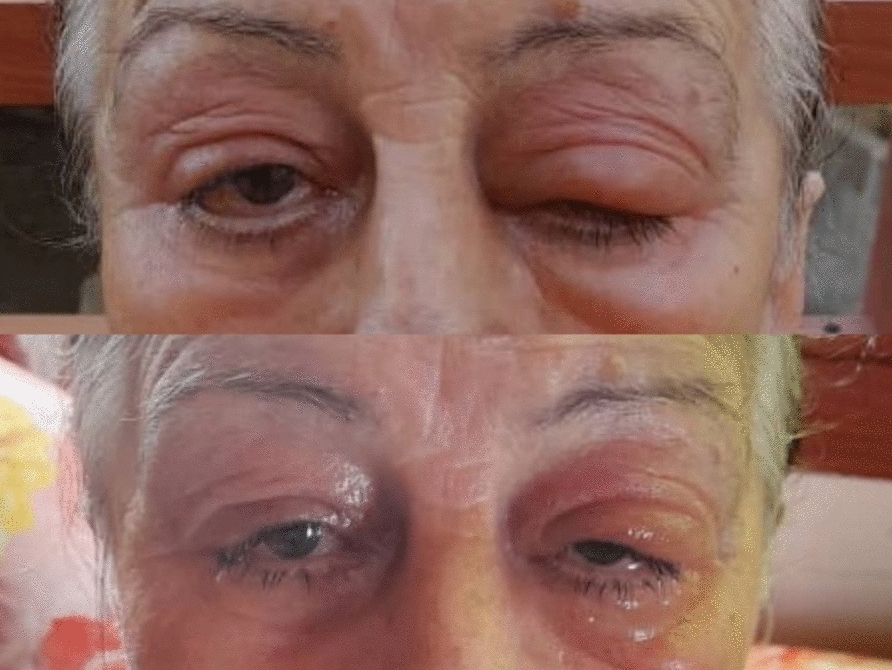
Clinical photograph demonstrating bilateral upper eyelid swelling with erythema and S-shaped deformity, consistent with lacrimal gland enlargement

On clinical examination, the patient was in stable general condition. Ocular examination revealed bilateral swelling and erythema of the lateral aspects of the upper eyelids. The patient reported excessive and persistent tearing, and an S-shaped deformity of both upper eyelids was observed with tender palpable fullness over the lacrimal gland. Best-corrected visual acuity was 20/25 in the right eye and 20/30 in the left eye. There was no ocular discharge or pain, and no other abnormalities were noticed (Fig. 1).

**Fig. 2:**
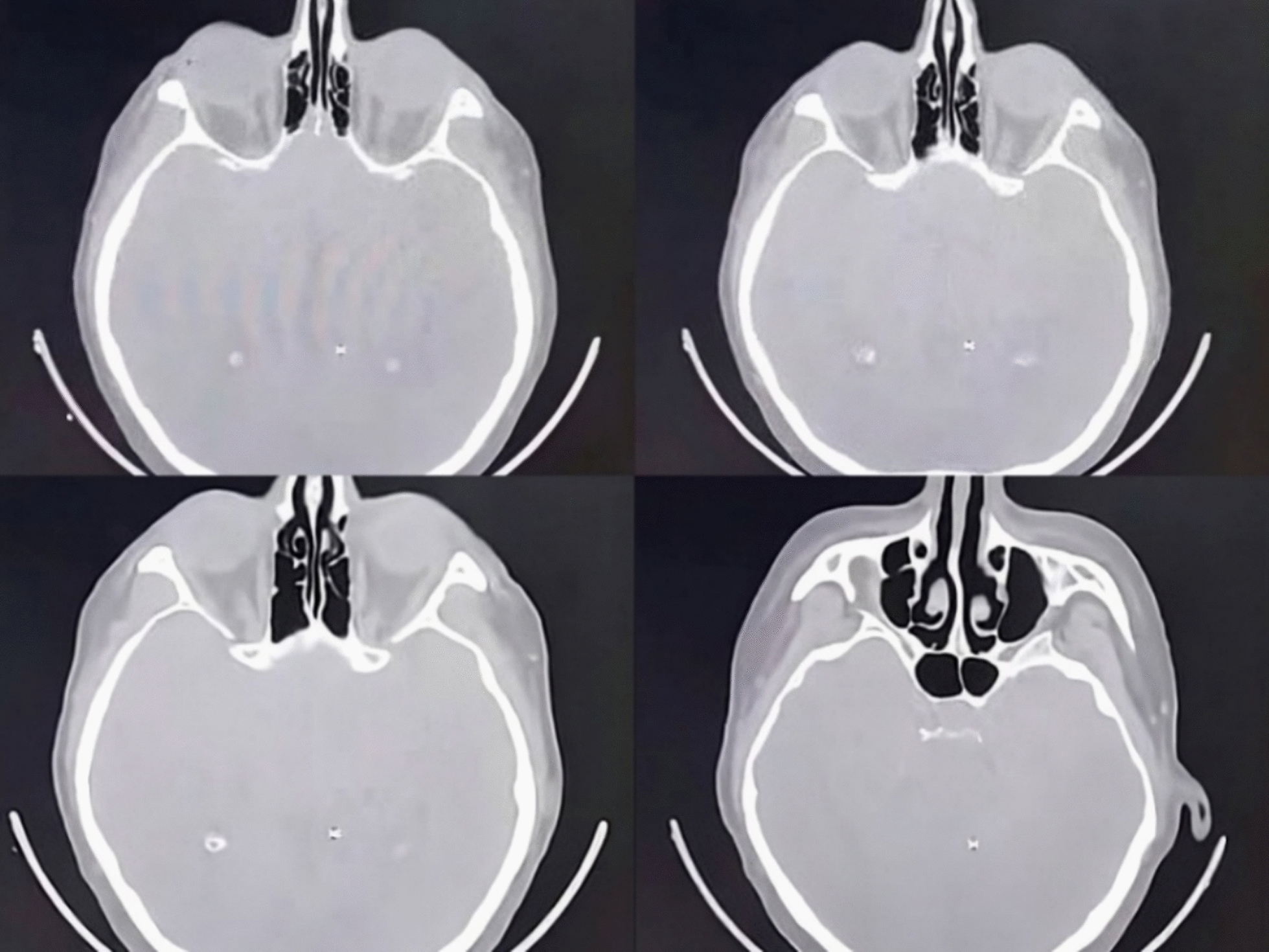
Axial computed tomography (computed tomography) scan of the head and orbits demonstrating unremarkable paranasal sinuses, with no evidence of mucosal thickening or abnormal opacification

Blood chemistry and urinalysis were undertaken to exclude systemic causes of fluid retention, including renal, hepatic, and thyroid disorders. The results were within normal limits, with no evidence of renal impairment, hepatic dysfunction, or thyroid abnormalities. Furthermore, the results did not indicate the presence of systemic infection or inflammatory changes. Computed tomography (CT) of the brain and orbits was conducted to rule out any paranasal sinusitis associations. The paranasal sinuses were unremarkable, with no evidence of mucosal disease or abnormal opacification (Fig. 2).

**Fig. 3:**
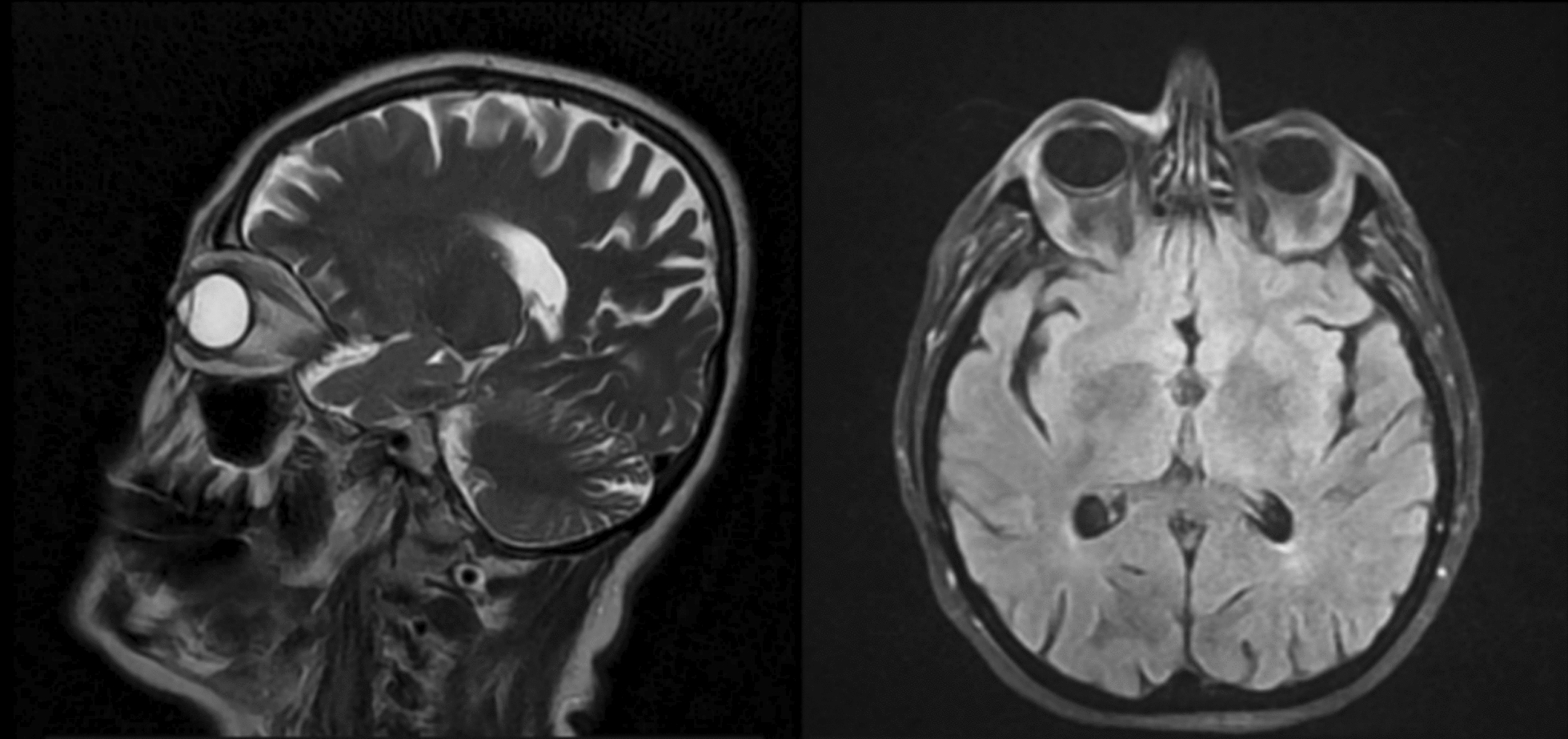
Head magnetic resonance imaging in axial and coronal planes demonstrating bilateral lacrimal gland enlargement with increased T2 signal intensity and homogeneous enhancement, consistent with dacryoadenitis

The clinical presentation raised suspicion of a viral etiology involving the lacrimal glands. Magnetic resonance imaging (MRI) demonstrated bilateral lacrimal gland enlargement with increased T2 signal intensity and homogeneous enhancement. The radiographic imaging scan revealed bilateral enlargement of the lateral and superior rectus muscles, including their tendinous insertions, with a pronounced left-sided proptosis. No focal mass lesion or infiltrative process was identified. Since dacryoadenitis may occasionally represent the only clinical sign of Epstein–Barr infectious mononucleosis, along with regional lymphadenopathy and preceding tonsillitis, these findings were suggestive of a possible Epstein–Barr virus-related acute dacryoadenitis, supported by upper eyelid edema and MRI-confirmed lacrimal gland involvement (Fig. 3).

Serological tests were requested in accordance, and the results demonstrated positivity for both Epstein–Barr virus viral capsid antigen IgM and IgG, consistent with a diagnosis of recent Epstein–Barr virus infection. Ultrasonographic imaging was also performed to evaluate for possible underlying abnormalities related to the EBV Infectious mononucleosis, demonstrating no features of organomegaly or other significant findings. After the final diagnosis, oral Prednisone was initiated at 1 mg/kg/day, maintained for 1 week, and tapered over the following 3 weeks. The patient showed marked clinical improvement with complete resolution of symptoms. At 6-month follow-up, the patient remained clinically stable and asymptomatic, with no evidence of recurrence.

## Discussion and conclusions

Acute dacryoadenitis has been attributed to a variety of pathogenic agents, with mumps being the most common prior to global immunization, as well as several bacteria such as staphylococci. Epstein–Barr virus (EBV) was first described as a causative agent of dacryoadenitis in the context of infectious mononucleosis in 1950 [7]. IM is classically found among children and young adult populations with 92% of individuals in their 20 s and 95% in their 40 s having contracted the virus [3, 8]. In our case, the patient developed dacryoadenitis due to a primary infection with Epstein–Barr virus at the age of 71.

The manifestations of EBV infection vary significantly by age. In children, it can be asymptomatic, while adolescents and adults typically present with classic symptoms, including general fatigue, cervical lymphadenopathy, tonsillar enlargement, and pharyngitis [9]. In contrast, in the elderly, the disease tends to follow more severe course with atypical symptoms such as jaundice, hepatomegaly, splenomegaly, and rashes. Approximately half of the elderly patients require hospitalization [10]. Complications of infectious mononucleosis (IM) may include splenic rupture, hepatitis, and airway obstruction, while ocular involvement is uncommon [11]. Acute dacryoadenitis has been documented only rarely as an ocular manifestation of Epstein–Barr virus infection [3]. Moreover, it has only been observed in some cases of children and young adults in particular [3]. In our case, the 71-year-old patient presented with simple classic symptoms of tonsillitis and lymphadenopathy without any atypical elders-related symptoms, and did not require any special care. To our knowledge, reports in older individuals appear to be extremely rare, and no previous cases have been documented in the literature to date.

EBV-associated dacryoadenitis typically presents as unilateral eyelid swelling, often painless or only mildly tender on palpation, usually without erythema. Marked redness, however, may indicate a secondary bacterial infection [3]. In this case, the patient exhibited bilateral eyelid swelling with marked erythema, in the absence of notable pain or signs of secondary bacterial infection. Chronic dacryoadenitis is generally seen on imaging bilaterally, while acute or sub-acute infectious dacryoadenitis is generally seen on imaging unilaterally [12]. Of clinical significance, the patient subsequently developed bilateral eyelid edema due to acute dacryoadenitis triggered by EBV infectious mononucleosis.

One study suggests that dacryoadenitis accompanied by lymphadenopathy may represent the sole indicator of EBV infection [2]. Acute dacryoadenitis usually presents among the early manifestations or precedes the onset of the typical symptoms of Epstein–Barr virus infection as reported [3]. Notably, our patient presented with only upper eyelid edema that developed after recovery from tonsillitis and lymphadenopathy 2 weeks earlier. Eyelid edema resulting from EBV-associated dacryoadenitis may obscure other clinical features and delay recognition, as illustrated in our case [2]. Distinguishing between eyelid edema and lacrimal gland swelling may be challenging, which may contribute to diagnostic uncertainty in patients with Epstein-Barr virus infection, and findings may be misinterpreted as isolated upper eyelid edema [3]. Furthermore, infectious mononucleosis should be included in the differential diagnosis of acute dacryoadenitis, as lacrimal gland involvement may predominate other systemic signs and symptoms, resulting in further delayed diagnosis [7]. Accordingly, the presence of eyelid edema as a prominent presenting feature prompted us to perform radiographic investigations to rule out any sinusitis associations.

Owing to the rarity of this presentation and the limited number of cases reported in the medical literature, a definitive treatment for EBV-associated dacryoadenitis has not yet been established [2]. The clinical manifestations of acute dacryoadenitis may suggest an infectious etiology, and in bacterial cases, the infection can extend to adjacent orbital tissues. Therefore, systemic antibiotic therapy is generally recommended as a first-line treatment when bacterial infection is suspected [13, 14]. EBV-associated dacryoadenitis is typically self-limited. Although systemic corticosteroids have been used in certain cases, their efficacy remains unclear. Most of the reported cases are steroid-responsive and showed clinical improvement, but routine administration is not universally recommended [2, 3]. In the present case, the patient developed progressive eyelid edema that was unresponsive to initial broad-spectrum antibiotic and corticosteroids therapy administered prior to establishing the final diagnosis and was managed with an extended 4-week corticosteroid course. It is important to recognize that persistent fever in a patient receiving antibiotic therapy may reflect an underlying viral infection rather than bacterial treatment failure, which could encourage unnecessary invasive treatment.

In summary, our case highlights an atypical presentation of Epstein–Barr virus infectious mononucleosis. The occurrence in an elderly patient, along with ocular manifestations at a later stage and the presence of bilateral dacryoadenitis, underscores the importance of maintaining a broad differential diagnosis and considering EBV infection in patients presenting with unexplained lacrimal gland enlargement, regardless of age or typical clinical expectations.

Methods: The work has been reported in line with the CARE 2017 criteria [15].

## Data Availability

No datasets were generated or analyzed during the current study.

## Abbreviations

EBV: Epstein–Barr virus
IM: Infectious mononucleosis
MRI: Magnetic resonance imaging
CT: Computed tomography

## References

[1] Dunmire SK, Verghese PS, Balfour HH. Primary Epstein-Barr virus infection. J Clin Virol. 2018;102:84–92. 10.1016/J.JCV.2018.03.001. 29525635 10.1016/j.jcv.2018.03.001

[2] Marchese-Ragona R, Marioni G, Staffieri A, De Filippis C. Acute infectious mononucleosis presenting with dacryoadenitis and tonsillitis. Acta Ophthalmol Scand. 2002;80:345–6. 10.1034/j.1600-0420.2002.800323.x. 12059880 10.1034/j.1600-0420.2002.800323.x

[3] Hayano S, Nakada N, Kashima M. Acute dacryoadenitis due to primary Epstein-Barr virus infection. Open Forum Infect Dis. 2022;9:1–3. 10.1093/ofid/ofac086. 10.1093/ofid/ofac086 PMC8962688 35355892

[4] Wang Y, Chen P. Research advances in infectious mononucleosis caused by Epstein-Barr virus. Open J Pediatr. 2024;14:108–21. 10.4236/ojped.2024.141012.

[5] Leung AKC, Lam JM, Barankin B. Infectious mononucleosis: an updated review. Curr Pediatr Rev. 2023;20:305–22. 10.2174/1573396320666230801091558. 10.2174/1573396320666230801091558 37526456

[6] Pflugfelder SC, Crouse CA, Atherton SS. Epstein–Barr virus and the lacrimal gland pathology of Sjögren’s syndrome. Adv Exp Med Biol. 1994;350:641–6. 10.1007/978-1-4615-2417-5_107. 8030548 10.1007/978-1-4615-2417-5_107

[7] Rhem MN, Wilhelmus KR, Jones DB. Epstein-Barr virus dacryoadenitis. Am J Ophthalmol. 2000;129:372–5. 10.1016/S0002-9394(99)00351-7. 10704555 10.1016/s0002-9394(99)00351-7

[8] Infectious mononucleosis - UpToDate. https://www.uptodate.com/contents/infectious-mononucleosis. Accessed 28 Jan 2026.

[9] Capelli M. Eyelid edema may be as a sign of dacryoadenitis in the course of Epstein-Barr Virus Infectious Mononucleosis. J Med Cases. 2023;14:208–12. 10.14740/jmc4114. 37435102 10.14740/jmc4114 PMC10332871

[10] Babiker HM, Wiedenbeck T, Robetorye RS, Acharya U, Wilansky S, Kusne S. Acute systemic viral infection masquerading as an infiltrating lymphoma in an elderly patient: a case report and review of the literature. Case Rep Med. 2013. 10.1155/2013/318358. 23476662 10.1155/2013/318358 PMC3583113

[11] Aburn NS, Sullivan T. Presenting with dacryoadenitis. Ophthalmology. 1996;103:776–8. 10.1016/S0161-6420(96)30617-9. 8637686 10.1016/s0161-6420(96)30617-9

[12] Ahn C, Kang S, Sa HS. Clinicopathologic features of biopsied lacrimal gland masses in 95 Korean patients. Graefes Arch Clin Exp Ophthalmol. 2019;257:1527–33. 10.1007/s00417-019-04327-w. 31025214 10.1007/s00417-019-04327-w

[13] Goold LA, Madge SN, Au A, Leibovitch I, McNab A, Tumuluri K, *et al*. Acute suppurative bacterial dacryoadenitis: A case series. Br J Ophthalmol. 2013;97:735–8. 10.1136/bjophthalmol-2012-302302. 23603486 10.1136/bjophthalmol-2012-302302

[14] Lam L, Pietris J, Quigley C, Davis G, Slattery J, Selva D. Bilateral sequential bacterial dacryoadenitis with abscess– a case report. BMC Ophthalmol. 2025. 10.1186/s12886-025-04063-2. 40251557 10.1186/s12886-025-04063-2 PMC12007365

[15] Gagnier JJ, Kienle G, Altman DG, Moher D, Sox H, Riley D. The CARE guidelines: consensus-based clinical case reporting guideline development. 10.1136/bcr-2013-201554. Accessed 22 Apr 2026.

